# Amino acid metabolic reprogramming in tumor metastatic colonization

**DOI:** 10.3389/fonc.2023.1123192

**Published:** 2023-03-14

**Authors:** Zihao Wang, Xingyun Wu, Hai-Ning Chen, Kui Wang

**Affiliations:** ^1^ Colorectal Cancer Center, West China Hospital, Sichuan University, Chengdu, China; ^2^ West China School of Basic Medical Science and Forensic Medicine, Sichuan University, Chengdu, China

**Keywords:** amino acid metabolism, metabolic reprogramming, cancer metastasis, distant organ colonization, metabolic targeting

## Abstract

Metastasis is considered as the major cause of cancer death. Cancer cells can be released from primary tumors into the circulation and then colonize in distant organs. How cancer cells acquire the ability to colonize in distant organs has always been the focus of tumor biology. To enable survival and growth in the new environment, metastases commonly reprogram their metabolic states and therefore display different metabolic properties and preferences compared with the primary lesions. For different microenvironments in various colonization sites, cancer cells must transfer to specific metabolic states to colonize in different distant organs, which provides the possibility of evaluating metastasis tendency by tumor metabolic states. Amino acids provide crucial precursors for many biosynthesis and play an essential role in cancer metastasis. Evidence has proved the hyperactivation of several amino acid biosynthetic pathways in metastatic cancer cells, including glutamine, serine, glycine, branched chain amino acids (BCAAs), proline, and asparagine metabolism. The reprogramming of amino acid metabolism can orchestrate energy supply, redox homeostasis, and other metabolism-associated pathways during cancer metastasis. Here, we review the role and function of amino acid metabolic reprogramming in cancer cells colonizing in common metastatic organs, including lung, liver, brain, peritoneum, and bone. In addition, we summarize the current biomarker identification and drug development of cancer metastasis under the amino acid metabolism reprogramming, and discuss the possibility and prospect of targeting organ-specific metastasis for cancer treatment.

## 1 Introduction

Metastasis is the main cause of cancer mortality, leading to more than 90% of cancer deaths (1). The occurrence of metastasis depends not only on the invasiveness of cancer cells, but also on the cells overcoming the obstacles caused by metabolic stress in the microenvironment of distant organs. Only a few cancer cells can overcome hypoxia, avoid apoptosis, escape from immune clearance, and finally adapt to the new microenvironment (2, 3). Therefore, metastasis is regarded as a rare event, only very few cancer cells with appropriate evolution of adaptability can successfully colonize at distant organs. Whether cancer cells with acquired metastatic characteristics are selected during invasion and colonization, or metastatic tendency has been marked in most cancer cells, metastatic cancer cells appear specific growth advantages of remote planting (4–6). The genetic factors and microenvironment causing selective pressures seem to confer these growth advantages. For example, several studies have shown that cancer cells elevated their antioxidant capacity to avoid being eliminated by the oxidative environment in the process of metastasis (7–10).

However, the metabolic reprogramming patterns of different metastatic cancers are not the same, indicating the metabolic heterogeneity of tumor metastasis. The complexity of microenvironment caused by different organ functions and needs of metastatic organs also determines the occurrence of tumor metastasis to some extent. Recently, growing evidence supports that under different selective stresses, tumor cells with specific metabolic advantages can selectively survive, even homologous primary tumor cells also show evolution of different metabolic phenotypes and vulnerabilities in various metastatic organs (11–15). These findings support the tendency of metastasis, that is, cancer will not metastasize randomly, but will spread to specific organs depending on tumor types. The intrinsic features of tumor and its interaction with host organ-specific microenvironment together determine the organ-specific metastatic behaviors of tumor cells (16). And this discrepancy of behaviors has been proved to stem from a complex set of metabolic reprogramming mediated by organ-specific gene expression (17–19). In addition to energy production and biosynthesis (20, 21), metabolic reprogramming also plays a central role in cell signal transduction (22, 23), epigenetic regulation (24, 25) and microenvironment adaptation (26–30).

As the building blocks of many biosynthesis, amino acids are involved in regulating many cellular processes such as biosynthesis of metabolic intermediating (31), energy production (32), epigenetic regulation (33) and cell signaling (34). There are nine out of twenty amino acids that cannot be synthesized, or the synthesis speed is far from meeting the needs in human beings and consequently must be supplied from food protein, called essential amino acids (EAAs, including lysine, tryptophan, phenylalanine, methionine, threonine, isoleucine, leucine, valine, and histidine). By contrast, the amino acids that can be synthesized *de novo* are called nonessential amino acids (NEAAs, including glycine, alanine, proline, tyrosine, serine, cysteine, asparagine, glutamine, aspartate, and glutamate). During cancer metastasis, amino acid metabolic reprogramming is commonly characterized as increased uptake of amino acids and augmented *de novo* synthesis of NEAAs. For example, cystine antiporter xCT (also known as SLC7A11) can potentiate secretion of glutamate to promote the invasive behavior of colorectal cancer (CRC) and breast cancer (35, 36). Although all amino acids are likely to contribute to the formation of metastasis, there are several amino acids emerged as main contributors and studied most, including glutamine, serine, glycine, branched chain amino acids (BCAAs), proline and asparagine. The metabolic reprogramming of these amino acids supports the metabolic dependence and vulnerability of cancer cells to adapt to the microenvironment of metastases.

## 2 Glutamine metabolism in tumor metastatic colonization

**Figure 1:**
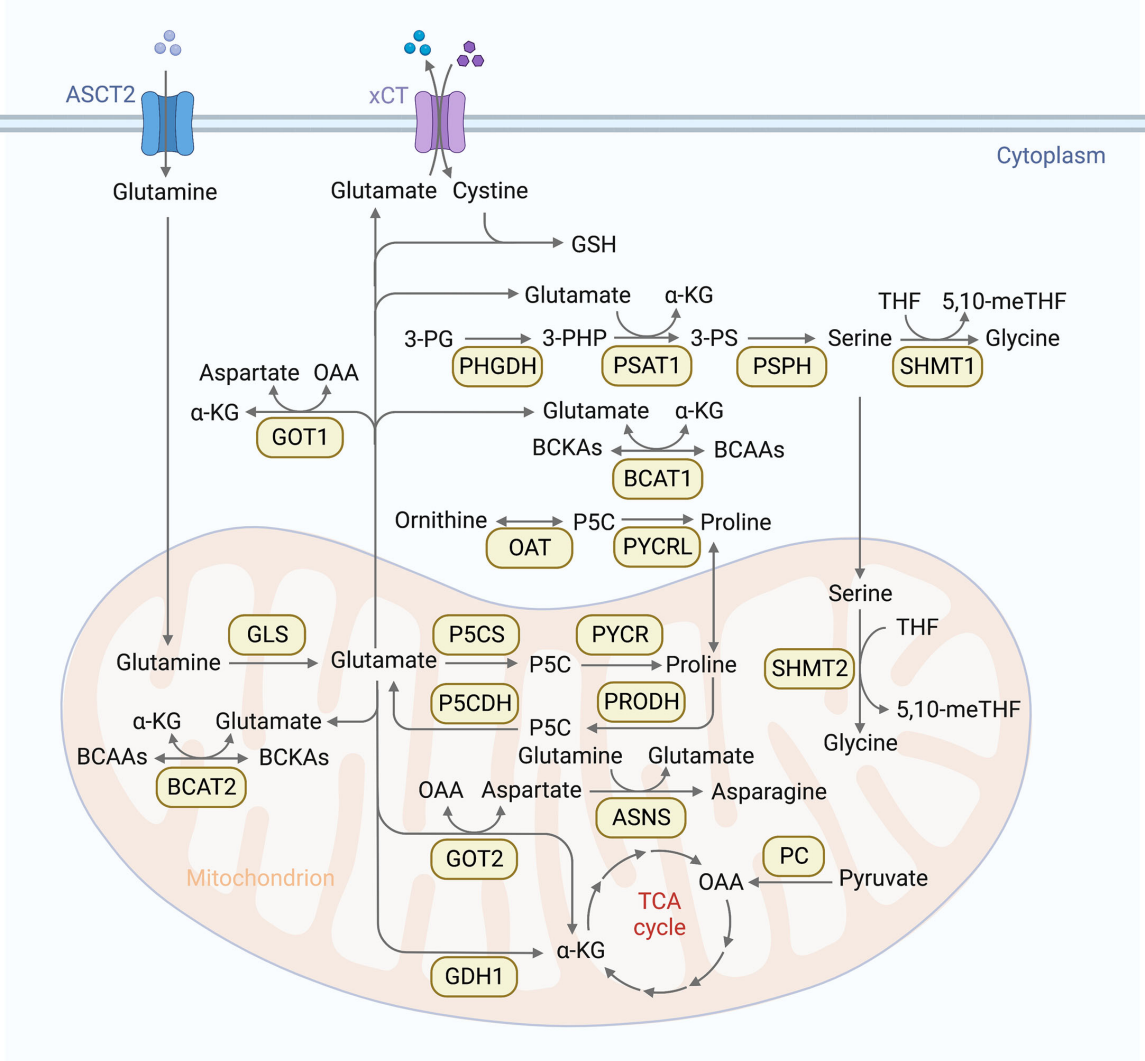
Overview of amino acid metabolic pathways. Cells absorb glutamine through the glutamine transporter ASCT, then glutamine is converted into glutamate through deamination catalyzed by glutaminase (GLS1/2) in mitochondria. Glutamate is further converted into α-ketoglutarate (α-KG) by glutamate dehydrogenase (GDH) in mitochondria. α-KG can promote oxidative TCA cycle and participate α-KG dependent reactions as cofactor. Another TCA supplement way is that pyruvate carboxylase (PC) catalyzes pyruvate into oxaloacetate (OAA). In addition, glutamate and OAA can be reversibly converted into asparagine and α-KG by glutamic-oxaloacetic transaminase (GOT1/2). Furthermore, glutamate can be secreted by cystine antiporter xCT to transport cystine into cells. Glutamate and cystine can produce glutathione (GSH) to rescue cancer cells from oxidative stress. Glutamine is also involved in serine metabolism. 3-phosphoglycerate (3-PG) is oxidized to 3-phosphate hydroxypyruvate (3-PHP) under the catalysis of phosphoglycerate dehydrogenase (PHGDH), and 3-PHP is catalyzed to 3-phosphoserine (3-PS) by phosphoserine aminotransferase 1(PSAT1), accompanied by the conversion of glutamate to α-KG. 3-PS is then dephosphorylated to serine by phosphoserine phosphatase (PSPH). Serine is reversibly catalyzed by serine-hydroxymethyltransferase 1 (SHMT1) to glycine in cytoplasm, or by SHMT2 in mitochondria. This reaction simultaneously converts tetrahydrofolate (THF) into 5,10-methylenetetrahydrofolate (5,10 methylene tetrahydrofolate), which is an important source of one carbon (1C) unit. Glutamine is involved in branched chain amino acids (BCAAs) metabolism as well. BCAAs can be catalyzed by BCAA transaminases (BCAT) to reversibly transfer nitrogen to α-KG to generate glutamate and branched chain ketoacid (BCKAs). Glutamine is also involved in proline synthesis. glutamate-γ-Semialdehyde (GSAL) can be synthesized from glutamate by Delta-1-pyrroline-5-carboxylate synthase (P5CS) or ornithine by ornithine aminotransferase (OAT), and then generates proline by pyrroline-5-carboxylic acid reductase (PYCR). PYCR can be converted back to Delta-1-pyrroline-5-carboxylic acid (P5C) by proline dehydrogenase/oxidase (PRODH/POX) in mitochondria. P5C can be metabolized to glutamic acid by pyrrolidine-5-carboxylate dehydrogenase (P5CDH) or ornithine by OAT. Glutamine can also be used as a nitrogen donor to participate in the reaction of aspartate to produce endogenous asparagine under the catalysis of asparagine synthetase (ASNS).

Glutamine is the most depleted amino acid in tumor cells (37). As an important metabolic fuel, glutamine meets the heavy demand of metastatic cells for ATP (38), biosynthetic precursors (39) and reductant (40). Therefore, the increased accessibility of glutamine strengthens the invasiveness of cancer cells and leads to distant metastasis. In addition, glutamine is the main source of carbon and nitrogen in cells. Catalyzed by a series of transaminases, glutamine is transformed to glutamate and provides amidogen for the synthesis of other nonessential amino acids. Therefore, as an important link in amino acid metabolism, glutamine metabolism can influence the synthesis of other amino acids. The first step of glutamine metabolism is the conversion of glutamine to glutamate. This deamination process is catalyzed by glutaminase (GLS1/2) in mitochondria. After that, glutamate is further catalyzed to α-ketoglutarate (α-KG) by glutamate dehydrogenase (GDH) with GDH1-catalyzed production of NADH and GDH2-catalyzed production of NADPH, concurrently. In glutamine-consuming cells, glutamate is the major source of α-KG. α-KG can feed the oxidative TCA cycle (41), and enable α-KG-dependent reactions as a co-factor (42). GDH1 can control the levels of α-KG to maintain the redox homeostasis of cancer cells (43). In addition, glutamate and oxaloacetate (OAA) can also be converted to aspartate and α-KG through the transamination of glutamic-oxaloacetic transaminase 2 (GOT2) in mitochondria. Whereas aspartate and α-KG can be converted back to glutamate and OAA by GOT1 in cytoplasm. Furthermore, glutamate can be secreted by cystine antiporter xCT to take in cystine. Glutamate and cystine can generate to glutathione (GSH) (**Figure 1**), an important antioxidant which protects cancer cells from the accumulation of reactive oxygen species (ROS) (44). Moderate ROS level can support tumor survival and proliferation in stressful microenvironment by activating related signal pathways. ROS accumulation will lead to serious damage to biomolecules, triggering cell death (45). Because of the dual roles of ROS in cancer progression, the effect of GSH in metastases is also complicated (45).

Because of the large demand for energy in metastatic cells and the insufficient efficiency of energy production in aerobic glycolysis, metastatic cells need energy supplement through other ways, especially the TCA cycle. As glutamate is the major source of α-KG, cancer cells rely on glutamine metabolism to produce α-KG to replenish TCA cycle (46). This process is called glutamine anaplerosis. Another TCA supplement way is that pyruvate, produced by aerobic glycolysis, generates OAA under the catalysis of pyruvate carboxylase (PC). Some studies have shown that, the differences in glutamine availability in the organ-specific microenvironment determine whether cancer cells prefer to PC-dependent anaplerosis or glutamine anaplerosis (28, 47). It has also been reported that breast cancer tends to rely on glutamine anaplerosis (48, 49), whereas lung cancer is more likely to show PC-dependent anaplerosis (47, 50, 51). Furthermore, this metabolic plasticity dominated by the availability of glutamine in organ-specific microenvironment has also been observed between metastatic and primary tissue samples in mouse models (52), supporting the function of glutamine metabolism reprogramming in metastasis.

### 2.1 Targeting glutamine metabolism in tumors

In view of the dependence and vulnerability of tumor cells on glutamine metabolism, several drugs have been developed to interfere with intracellular glutamine catabolism. For example. the GLS1 inhibitor BPTES, could decreased the proliferation and migration of colorectal cancer (CRC) cells (53). And the glutaminase inhibitor CB839 could inhibit the proliferation of triple negative breast cancer cells, which is related to the significant reduction of glutamine consumption, glutamate production, and GSH levels (54). In addition, some studies have found that tumor cells could promote the glutamine synthesis and metabolism in cancer-related fibroblasts (CAFs), while CAF-generated glutamine could support the metabolism and invasion of cancer cells in a symbiotic manner (55). Furthermore, simultaneous targeting of glutamine anabolism in CAFs and glutamine catabolism in cancer cells could inhibit the growth and metastasis of ovarian tumors in an orthotopic intra-ovarian mouse model (56). Additionally, targeting glutamine metabolism could inhibit the generation and recruitment of myeloid-derived suppressor cells, leading to an increase of tumor-associated macrophages, thereby inhibiting the occurrence and development of tumors. This study suggests that targeting glutamine metabolism makes tumors susceptible to immunotherapy (57).

### 2.2 Lung metastasis

Lung is a common site for metastases formation of various malignant tumors. Because glutamine is largely depleted for energy supply and biosynthesis in metastatic cancer cells, the glutamine level is generally low in the microenvironment of metastases, with no exception in lung metastases. For example, in a mouse model of spontaneous metastasis of Lewis lung cancer (LLC), the content of glutamine in lung metastases was reduced by ~ 60% compared with the primary tumor (58). It was also reported that the suppression of glutamine transporter ASCT2 (also known as SLC1A5) decreased glutamine uptake *in vitro* and significantly inhibited tumor growth and metastasis *in vivo* (59). Moreover, when cultured in low glutamine conditions, CAFs migrated towards glutamine driven by glutamine dependence, and in turn facilitated the migration of tumor epithelial cells (55). These findings point the rapidly depletion and heavily dependence on glutamine of metastatic cancer cells.

In addition to glutamine itself, glutamine metabolic pathway can also regulate multiple cellular signals to promote the formation of lung metastases. GLS1 is the key enzyme hydrolyzing glutamine into glutamate. The upregulated level of GLS1 in CRC cells could promote hypoxia-induced migration and invasion, leading to metastatic colonization in lung (60). Another key enzyme is GDH1, which converts the glutamate to α-KG. It was found that the α-KG produced by GDH1 could bind to CamKK2 and activate AMPK, which contributes to anoikis resistance and pro-metastatic signals in LKB1-deficient lung metastasis (61).

**Figure 2:**
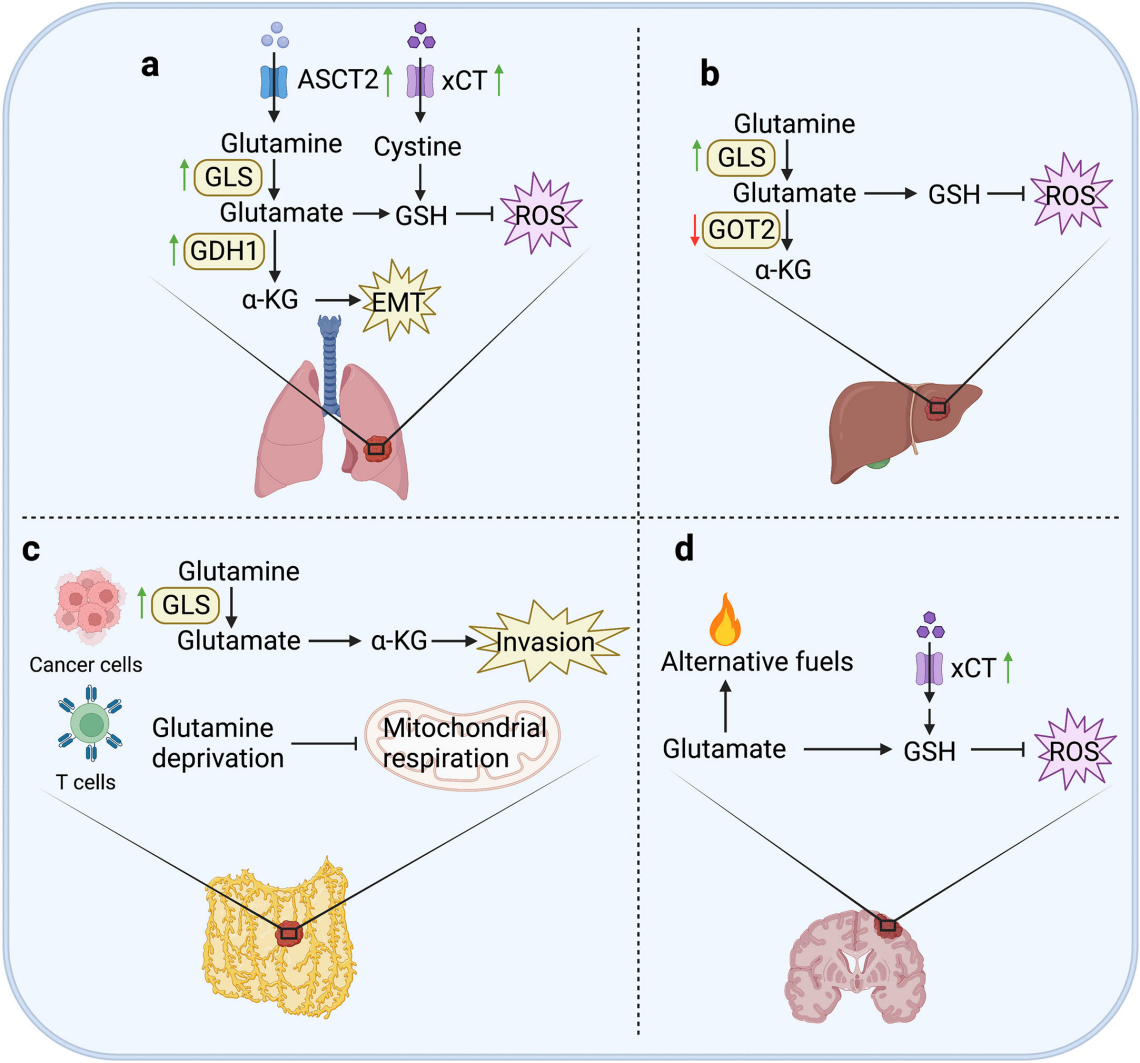
Glutamine metabolism in tumor metastases. **(A)** In lung, the expression of ASCT2 can maintain the intracellular glutamine level by seizing in the microenvironment. Upregulation of GLS1 in CRC cells can enhance glutamine metabolism to promote the formation of lung metastases. The expression of GDH1 contributes to anoikis resistance and pro-metastatic signals in LKB1-deficient lung metastasis. xCT can promote cystine uptake to increase the generation of GSH to constrain ROS levels, and enhance the invasiveness of breast cancer cells. **(B)** In liver, the expression of GLS1 can generate GSH to attenuate ROS accumulation. However, knockdown of GOT2 can promote hematogenous and intrahepatic metastasis of HCC cells. **(C)** In peritoneum, upregulated GLS1 expression is closely related to high invasiveness of OvCa cells. Moreover, glutamine metabolism can perturb T cell mitochondrial respiration to induce immune evasion of OvCa cells. **(D)** In brain, glutamine can be used as an alternative fuel to avoid energy deficiency in metastatic cells. In addition, the expression of ASCT2 is upregulated in human glioma cells, and overexpression of xCT can resist oxidative stress to promote metabolic adaptation of brain metastasis in breast cancer cells.

As the main organ of human respiration, lung is not only exposed to high levels of oxygen, but also filter the toxic waste in the systemic circulation. High levels of oxygen and toxic compounds lead to increased oxidative stress, which is a unique microenvironment for the colonization of lung metastatic cells. Lung metastatic cells must find ways to resist oxidative damage. Therefore, metastatic cancer cells require the enhanced uptake of cystine and glutamate to generate to GSH as a reductant to control ROS levels. Evidence indicates that cystine antiporter xCT could enhance the invasiveness of breast cancer cells by promoting cystine uptake (**Figure 2**). Immunotargeting xCT delayed established subcutaneous tumor growth of breast cancer in mice, and anti-xCT antibody severely impaired the formation of lung metastases (36).

### 2.3 Liver metastasis

Interestingly, although glutamine metabolism supports the occurrence of liver metastases, it was reported that knockdown of GOT2 promoted migration and invasion of HCC cells, as well as hematogenous and intrahepatic metastasis in HCC mouse models. Mechanistically, loss of GOT2 expression could reprogram glutamine metabolism to enhance glutamine decomposition, nucleotide synthesis, and glutathione synthesis (62). Oxidative stress is also considered to be a possible cause of chronic liver injury and cancer development (63). Therefore, in liver metastases, oxidative stress is ubiquitous and affect the invasiveness of cancer cells. It was reported that in melanoma cells, GSH content and metastatic activity showed a significantly positive correlation (64). However, targeting GLS1 significantly attenuated the stemness properties in HCC by promoting ROS accumulation and inhibiting Wnt/β-catenin pathway (65) (**Figure 2**). Indeed, the effect of GSH on liver metastases depends on whether the generation of GSH causes ROS accumulation. For example, the increased GSH level could promote metastasis of MHCC-97H cells (TP53, R249S), but inhibit metastasis of SMMC-7721 cells (TP53 wildtype) (66).

### 2.4 Peritoneum metastasis

Peritoneal metastasis is usually from intraperitoneal organs. The metastases are always accompanied by nutritional deficit, characterized by increased resting energy expenditure, muscle mass loss and protein catabolism (67). After cancer cells falling into the peritoneal cavity, reprogramming of glutamine metabolism is required to compensate ineffective oxidative phosphorylation in mitochondria to resist anoikis (67). Upregulated GLS1 expression was observed in high-invasive ovarian cancer (OvCa) cells, which are more dependent on glutamine than the low-invasive cells. In addition, glutamine deprivation suppressed the invasiveness of OvCa cells through activating STAT3, which could be restored by α-KG supplementation (68). Moreover, OvCa could evade immune control by perturbing T cell glutamine metabolism and effector function (**Figure 2**). It has been reported that malignant ascites fluid obtained from OvCa patients could regulate the abundance of glutamine carriers to limit the glutamine influx, resulting in reduced mitochondrial activity in XBP1-deficient T cells (69).

### 2.5 Bone metastasis

Bone metastasis can come from a variety of malignant tumors originating outside bone tissues, among which breast cancer is the most likely cancer type to have bone metastasis. Bone metastasis also showed different metabolic characteristics from the primary sites. A previous study demonstrates that bone metastatic breast cancer cells were more dependent on glutamine than parental cells. Interestingly, even in the environment of high glucose level, breast cancer cells still showed dependence on glutamine to enable their survival (70). This study supports a unique glutamine metabolic pattern of bone metastases in breast cancer compared with primary sites.

### 2.6 Brain metastasis

As the organ with the highest energy demand, the brain consumes one fifth of the energy produced by glucose (71). At the same time, in order to avoid energy deficiency, the brain has metabolic plasticity and can use other nutrients as energy sources. For example, the brain can use acetate, ketone bodies or short and medium chain fatty acids to supplement energy supply when glucose becomes limiting, such as low blood glucose (72). Brain metastatic tumor cells have also shown the ability to obtain energy from non-glucose sources. Some studies have observed that brain metastases exhibit metabolic plasticity and flexibility by using glutamine and branched chain amino acids as alternative fuels (73, 74).

Besides, glutamate also acts as a synaptic excitatory neurotransmitter in the brain. Glutamate is released into the synaptic space through exocytosis, and is absorbed into astrocytes by glutamate transporters after the completion of neurotransmission. Glutamate is metabolized by glutamine synthetase (a glia-specific enzyme) to synthesize glutamine (75, 76). Then glutamine is converted back to glutamate by the neuron-specific glutaminase in neurons, and continues to circulate in synaptic transmission, called Gln/Glu cycle (GGC) (77–79).

Anyhow, brain metastases demonstrate significant dependence on glutamine metabolism. Actually, brain cancer cells always show stronger competitiveness for glutamate than normal cells by upregulating the expression of glutamate importers. There are studies showing that the expression of ASCT2 was upregulated in human glioma cells (80, 81). In addition, the overexpression of xCT could promote the formation of brain metastases through promoting cystine uptake and glutamine oxidation (**Figure 2**), indicating that glutamine metabolism contributes to the metabolic adaptation of brain metastasis regulating redox homeostasis (52).

## 3 Serine and glycine metabolism

Serine and glycine are another two amino acids required in large quantities during metastasis to feed up biosynthesis such as glycolysis, glutathione and nucleotide production (82). In addition to exogenous uptake of serine and glycine, the serine synthesis pathway (SSP) is commonly hyperactivated in metastatic cancer cells (83–85). The SSP includes two processes: the *de novo* synthesis of serine from the glycolysis branch, and the conversion of glycine to serine. Serine is converted from 3-phosphoglycerate (3-PG, an intermediate product of glycolysis) continuously catalyzed by phosphoglycerate dehydrogenase (PHGDH), phosphoserine aminotransferase 1 (PSAT1), and phosphoserine phosphatase (PSPH). Firstly, 3-PG is converted to 3-phosphate hydroxypyruvate (3-PHP) by the catalysis of PHGDH. Secondly, 3-PHP is catalyzed to 3-phosphoserine (3-PS) by PSAT1, accompanied by the conversion of glutamate to α-KG. Thereafter, 3-PS is dephosphorylated by PSPH to produce serine. After exogenous uptake or *de novo* serine synthesis, serine is facilitated into glycine by serine-hydroxymethyltransferase (SHMT1/2), with SHMT1 reversibly catalyzing the reaction in the cytoplasm or SHMT2 in the mitochondria (86). This reaction converts tetrahydrofolate (THF) to 5,10 methylene tetrahydrofolate (5,10-meTHF) at the same time, providing a carbon unit for the nucleotide synthesis (**Figure 1**). The flow direction of the reversible reaction depends on the supply and demand of one carbon (1C) units in each compartment. In massively and rapidly synthesized metastatic cancer cells, 1C units are mainly derived from mitochondria. Therefore, SHMT2 was associated with tumor invasiveness in multiple cancer types and could predict the prognosis of patients with lung metastases from breast cancer (87–92). However, the specific role of SHMT1 in metastasis seems to depend on the type of cancer (93, 94).

### 3.1 Targeting serine and glycine metabolism in tumors

In view of the importance of serine and glycine metabolism for the occurrence and development of tumors, key enzymes in the serine and glycine metabolism may become powerful clinical markers and therapeutic targets for cancer. It has been reported that serine and glycine metabolism can regulate metabolic flux to influence tumor heterogeneity and drug resistance (95). Furthermore, intervention in the metabolism of serine and glycine was found to be an effective and less toxic method to improve the prognosis of patients with MYCN-amplified neuroblastoma (96, 97). A recent clinical trial demonstrated that high CDK12 status could indicate a significant reduction in the distant metastasis rate of chemotherapy group and non-chemotherapy group, and integrative transcriptomic and metabolomic analysis revealed that the hyperactivation of serine-glycine-carbon network is an inherent metabolic feature of CDK12-induced tumors (98). Targeting the key enzymes in serine and glycine metabolism may be an effective treatment strategy. It was reported that PHGDH inhibitors could reduce brain metastasis (99). In addition, treatment of triple-negative breast cancer with adriamycinin could activate SSP, resulting in an increased production of antioxidant GSH, and then resist adriamycinin-induced ROS. However, the PHGDH specific inhibitor NCT-503 significantly debilitated the adriamycinin resistance of triple-negative breast cancer, suggesting that the synergistic treatment on inhibition of key enzymes in serine and glycine metabolism could be a more effective treatment strategy for drug resistance (100).

### 3.2 Lung metastasis

**Figure 3:**
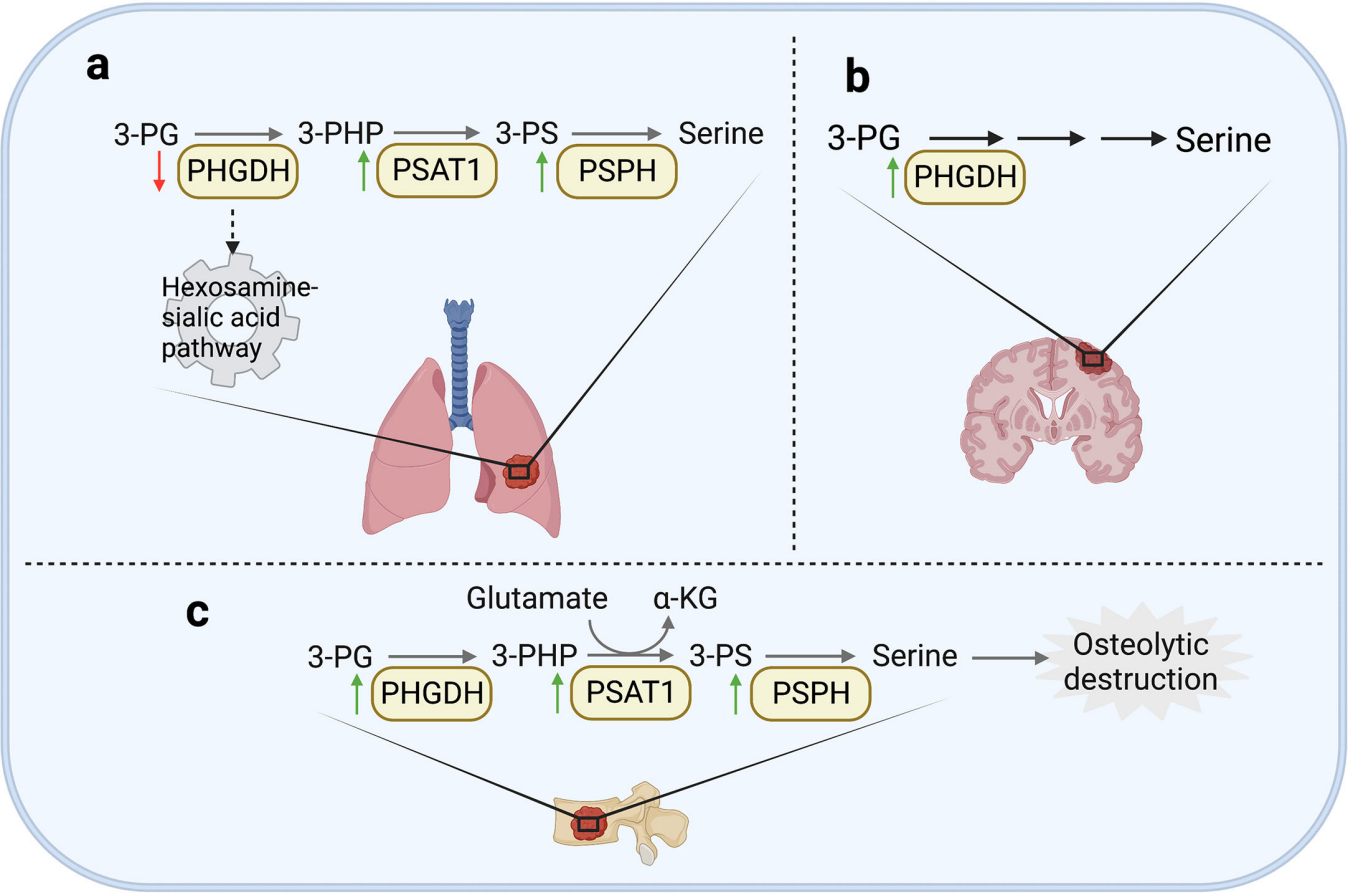
Serine and glycine metabolism in metastases. **(A)** In lung, the enhanced expression of PSAT1 was widely observed in various cancer tissues. In addition, PSPH is also observed to be upregulated in lung metastasis of NSCLC cells and melanoma cells. However, the low expression of PHGDH can enhance cell migration and invasion in breast cancer by activating hexosamine sialic acid pathway. **(B)** In brain, PHGDH can promote formation of brain metastases by enhancing serine synthesis. **(C)** In bone, PSPH, PSAT1 and PHGDH are all upregulated in bone metastases of breast cancer, resulting in osteolytic destruction.

It has been widely studied that exogenous serine uptake and endogenous serine synthesis both functionally support the progression of various cancers (90, 99, 101). In lung metastases, the specific oxidative pressure in the microenvironment and the demand for synthetic materials result in the dependence on SSP (31, 102). Increasing evidence shows that the expression of enzymes involved in SSP is related to lung metastasis in various cancers. For example, the overexpression of PSAT1 promoted the invasion of lung cancer cells *in vitro* and the metastatic lung colonization capacity *in vivo* (103). Recent work shows that in lung adenocarcinoma, PSAT1 overexpression could contribute to erlotinib resistance and tumor metastasis. Mechanistically, the activation of metabolic activity of PSAT1 could inhibit ROS-dependent JNK/c-Jun pathway to inhibit apoptosis. PSAT1 could also interact with IQGAP1 and then activate STAT3 to promote migration independent of its metabolic activity (104). Another essential enzyme is PSPH, which was also observed to be upregulated in lung metastases. It was reported that increased expression of PSPH promoted the invasiveness and metastatic potential of non-small cell lung cancer (NSCLC) cells by activating MAPK signaling pathways (105). Moreover, PSPH could induce a decrease of 2-hydroxyglutarate level and then enhance histone methylation modification to promote tumor growth and lung metastasis of melanoma cells (106). An interesting point is the heterogeneity of PHGDH in primary and metastatic tumors in breast cancer. Although the high expression of PHGDH supported the proliferation of primary tumors by its catalytic activity, PHGDH at low protein level non-catalytically enhanced the formation of metastases. Mechanistically, the loss of PHGDH released the glycolytic enzyme phosphofructokinase (PFK) and activated the hexosamine-sialic acid pathway, leading to aberrant protein glycosylation, thereby potentiating cell migration and invasion (107) (**Figure 3**). It was also found that the silencing of PHGDH under hypoxia conditions could lead to the decrease of NADPH level, disturb mitochondrial redox homeostasis, and promote apoptosis, resulting in the abrogation of breast cancer stem cells enrichment and lung metastases formation (108). Interestingly, although SHMT2 was observed as an oncogenic protein in various cancers, a study showed that SHMT1 inhibited the lung metastasis of HCC cells by suppressing the production of ROS (109).

### 3.3 Brain metastasis

In the microenvironment of brain metastases, the content of serine and glycine is significantly lower than that of plasma (110, 111), which might be caused by the huge demand for serine and glycine of brain metastasis tissues (112). It has been demonstrated that PHGDH could promote nucleotide synthesis and cell growth in highly aggressive brain metastatic cells by promoting SSP (**Figure 3**). Genetic silencing or pharmacological inhibition of PHGDH alleviated brain metastasis as well as prolonged the overall survival time of mice, but did not inhibit the growth of extracranial tumors. These results indicate that the availability of amino acids in the brain metastasis microenvironment determines the dependence of serine synthesis pathway (99).

### 3.4 Bone metastasis

As previously mentioned, bone metastasis of breast cancer shows glutamine-dependent metabolic transformation. This dependence is related to the promotion of glutamine utilization by SSP. As previously stated, regardless of the glucose concentration, glutamine is critical to the proliferation of bone metastasis cells from breast cancer. In terms of mechanism, the decreased expression of kinase C zeta (PKC-ζ) in bone-derived breast cancer cells led to the upregulation of PHGDH, PSAT1 and PSPH, consequently promoted the utilization of glutamine through serine biosynthesis (70). It has also been reported that the expression of PSPH, PSAT1, PHGDH, and serine transporter SLC1A4 was unregulated in bone metastasis by comparing the genome-wide gene expression profile between the highly bone metastatic variant MDA-MB-231 (SA) and its parental cell line MDA-MB-231 in mice models (**Figure 3**). And in primary breast cancer, the upregulation of PHGDH and PSAT1 is also significantly related to the shortening of overall survival time and the malignancy of breast cancer (113). Mechanistically, serine is an essential factor for differentiation of mesenchymal bone marrow precursors into osteoclasts (114), which may lead to a vicious circle of osteolytic bone metastasis (113).

## 4 Branched chain amino acids metabolism

Leucine, isoleucine, and valine are three branched chain amino acids (BCAAs), which have similar chemical and metabolic properties. As essential amino acids for human, BCAAs cannot be synthesized *in vivo*, so the metastases must obtain them from the circulation or surrounding tissues. However, their catabolism is highly reversible. BCAA catabolism means that compartment specific BCAA transaminases (BCAT1/BCAT2) transfer nitrogen from free BCAAs to α-KG to generate glutamate and its corresponding branched chain ketoacid (BCKA). BCAT1 catalyzes the reaction in the cytoplasm, while BCAT2 in the mitochondria (**Figure 1**) (115). BCAA catabolism can meet several important requirements for cancer cells. Firstly, the decomposition of BCAAs can provide carbon for the synthesis of other molecules. The catabolism of BCAAs can provide energy for cells by promoting TCA cycle and oxidative phosphorylation. Secondly, BCAAs can provide nitrogen for *de novo* synthesis of nucleotide and amino acid as well. Thirdly, BCAA metabolism can influence the levels of metabolite-derived cofactors which is important for epigenic modification. Finally, BCAAs can affect protein synthesis by acting as raw materials for synthesis or signaling molecules for uncovering nutritional status of cells (116). For example, it was observed that in breast cancer, BCAA catabolism was specifically activated in high metastatic potential cell lines (117). Previous studies have also shown that BCAT could support cell invasion in various cancers, such as lung cancer and breast cancer (118, 119).

Because BCAAs must be ingested by diet, the gastrointestinal tract is the main source of BCAA. The gastrointestinal tract can hydrolyze the complex polypeptide containing BCAAs to the dipeptide/tripeptide of BCAAs. BCAAs are mainly absorbed in the small intestine and transported to the liver through the portal vein (120), which may be the reason why few studies have reported that liver metastasis is dependent on BCAAs compared with other metastasis sites.

### 4.1 Targeting BCAA metabolism in tumors

In 2013, Tonjes et al. (121) reported the overexpression of BCAT1 in glioma, which attracted the cancer research field to start the in-depth study of BCAA metabolism in cancer. In the following years, BCAT1 was gradually identified as an important prognostic cancer marker. It was found that BCAT1 was overexpressed in metastatic tissues of cancer patients (118), supporting the dependence of metastasis on BCAA metabolism

It was reported that although the pancreatic ductal adenocarcinoma (PDAC) and NSCLC are both driven by Kras activation and Trp53 deletion, they showed different BCAAs dependence. The BCAA catabolic enzyme expression in NSCLC tumors was higher than the PDAC tumors both in in mouse models and human tissues. The BCAT deletion could inhibit the formation of NSCLC tumors, but not PDAC tumors (122), suggesting that cancer metabolic dependence depends on the origin tissue. However, another study demonstrated that BCAT2 knockout in ME2-deficient PDAC cell lines inhibited cloning, which could be rescued by nucleotide supplementation (123), suggesting that gene mutation also affects the effect of BCAA metabolism on cancer progression. Therefore, cancer cells show heterogeneous dependence on BCAA metabolites. In order to successfully develop therapeutic strategies targeting BCAA metabolism, it is necessary to clarify the driving mechanism of BCAA metabolism on cancer cells in future research.

### 4.2 Lung metastasis

**Figure 4:**
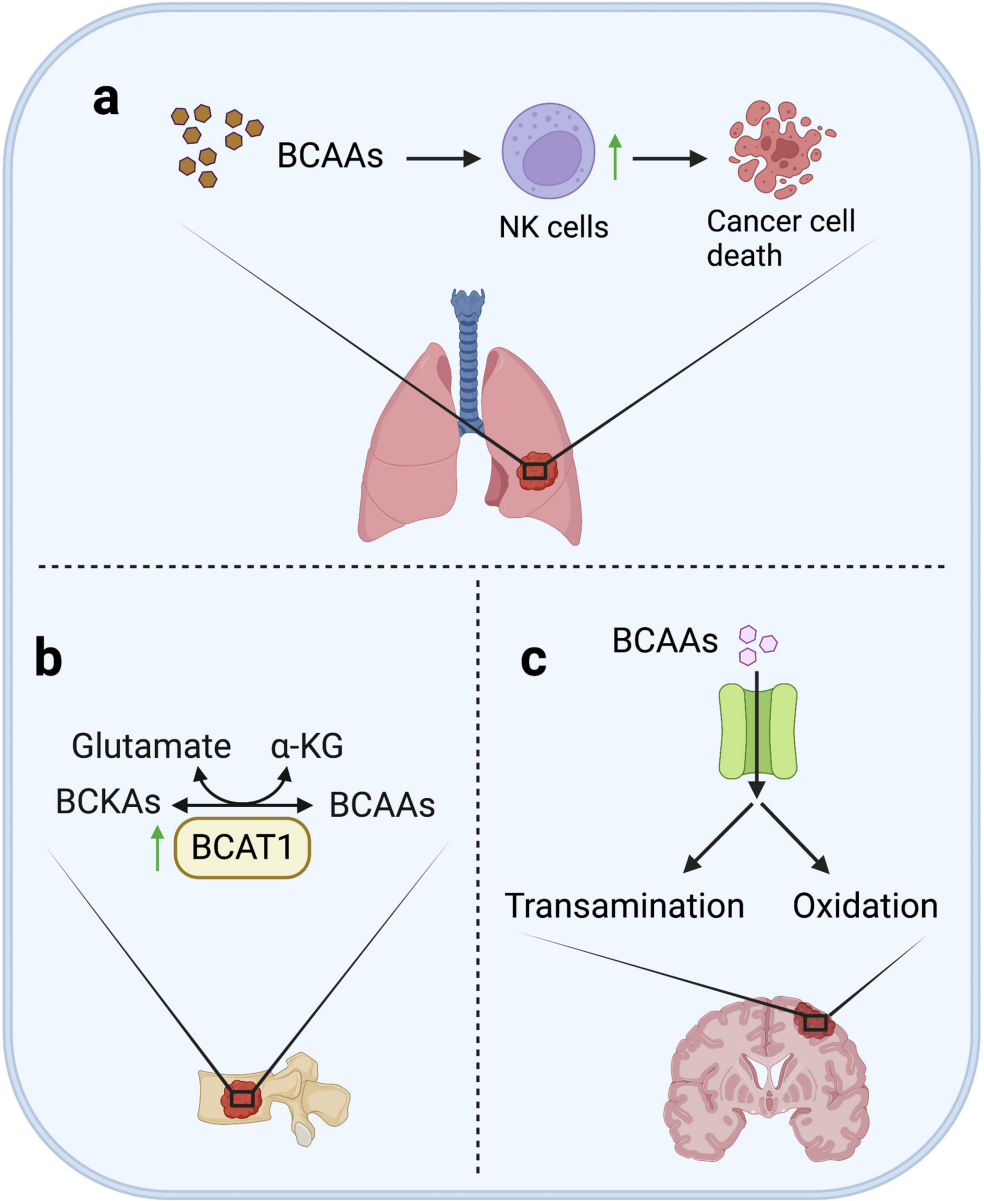
BCAAs metabolism in metastases. **(A)** In lung, although BCAAs are essential in supporting the proliferation and growth of various cancer, high level of BCAA may not promote formation of lung metastases, but on the contrary, high BCAA can inhibit lung metastasis of breast cancer by activating the tumor infiltrating-NK cells. **(B)** In bone, increased BCAT1 transcription can enhance BCAA catabolism to promote bone metastasis through remedy of α-KG depletion in lung cancer cells. **(C)** In brain, BCAAs are ingested in large quantities, and then transaminated to glutamate or oxidated for energy supply.

Although BCAA catabolism is essential in supporting the proliferation and growth of various cancers, high BCAAs levels seem to activate the immunosurveillance in lung metastases at the same time, and eventually result in suppression on lung metastasis. It was reported that in breast cancer mouse models, less lung metastases were observed in the high BCAAs diet-fed mice. The mechanism is that high levels of BCAAs activated tumor-infiltrating NK cells in lung metastases (124). This study indicates that high BCAA may not promote the proliferation of cancer cells in lung metastases, but conversely inhibit lung metastasis by promoting the activity of tumor-infiltrating NK cells in lung metastases (**Figure 4**).

### 4.3 Brain metastasis

Because of the high demand of brain for glutamate, cells need an amino group donor that is readily transaminated to maintain the high anabolism of glutamate. Actually, at least two thirds of brain glutamate are generated from BCAAs (125). Consequently, a large number of neutral amino acid transporters is highly expressed in cerebral vascular endothelial cells to support the continuous and large-scale uptake of BCAAs (126). It was reported that, using glucose analogue tracer ^18^FDG and ^11^C-BCAA tracer for brain metastasis imaging, ^11^C-BCAA was found to exhibit higher sensitivity (127–130). In addition, increased BCAAs oxidation was observed in two kinds of brain metastatic cancer cells (MDA-MB-231Br3 and MDA-MB-361) that are different in origin (73). All above studies support the dependence of brain metastasis on BCAAs.

### 4.4 Bone metastasis

Enhanced BCAA catabolism is important for bone metastasis. Previous studies have shown that increased BCAT1 transcription is associated with α-KG depletion and high expression of SOX2, promoting bone metastasis of lung cancer cells, while knockdown of BCAT1 could reduce cell migration *in vitro* and metastasis *in vivo* (**Figure 4**) (118). This result supports that enhanced BCAA catabolism can promote metastasis by maintaining energy supply and activating stemness-related pathways of metastatic cells.

## 5 Proline metabolism

The reprogramming of proline metabolism plays a crucial role in the development of cancer. Proline metabolism is involved in ATP production, biological synthesis of macromolecules (including protein and nucleotide synthesis), and redox homeostasis in tumor cells (131–134). Proline metabolic pathways consist of proline synthesis and catabolism. As for proline synthesis, the immediate precursor glutamate-γ-semialdehyde (GSAL) can be generated from ornithine by ornithine aminotransferase (OAT) in cytoplasm, or from glutamate by delta-1-pyrroline-5-carboxylate synthase (P5CS) in mitochondria. Then, newly produced GSAL can automatically transform to delta-1-pyrroline-5-carboxylic acid (P5C). Finally, pyrroline-5-carboxylic acid reductase (PYCR) catalyzes P5C to proline with the concurrent oxidation of NADH in mitochondria or NADPH in cytoplasm, while proline can be converted back to P5C by proline dehydrogenase/oxidase (PRODH/POX) in mitochondria (135). P5C can also be catalyzed to glutamate by pyrrolidine-5-carboxylate dehydrogenase (P5CDH) or ornithine by OAT (**Figure 1**) (136–138).

Because of the dependence of metastases on energy supply and biosynthesis, proline metabolism, which is closely related to glutamine metabolism, is also extremely essential in the formation of metastases. It was reported that PRODH, the key enzyme for proline degradation, could support growth of breast cancer cells in 3D culture, and promote the formation of lung metastasis in mice models. In particular, compared with primary breast cancer in patients and mice, the expression of PRODH and proline catabolism in metastatic tumors were increased (139). In several mouse models of metastatic breast cancer, inhibition of PRODH could inhibit the formation of metastases without adverse effects on normal cells (139). Furthermore, it was observed that chromatin remodeling factor LSH activated PRODH to decrease proline level to induce EMT in NSCLC (140), proving that PRODH could rescue the metastatic cancer cells from the nutrient stress through enhanced proline catabolism. Additionally, PYCR1 expression in human breast cancer has also been found to be associated with invasion (141). In short, the inhibition of proline metabolism by targeting key enzymes has become a potential strategy to interfere with metastasis formation.

Another important flow direction of synthetic proline is to produce collagen, the major component of extracellular matrix (ECM), by prolyl-4-hydroxylases (P4HA) in the endoplasmic reticulum (134). ECM mediates the interaction between cancer cells and stromal cells and promotes the colonization of metastatic cells (142). For example, ECM density has also been proved to be the main barrier for T cells infiltrating tumor beds in human NSCLC (143). Moreover, cancer cells could reshape the extracellular matrix of the metastatic niche to promote the colonization of their own metastases through hydroxylation of collagen, which is activated by elevated P4HA (144). In addition, a research showed that HIF-1 can promote the expression of P4HA to promote cancer cell alignment along collagen fibers, and consequently enhance invasion and metastasis to lymph nodes and lungs (145). These studies support that collagen synthesis could promote tumor invasion and metastasis by regulating cell adhesion and migration, biological signal transduction, and the growth of tissues and organs. Although proline metabolism has been proved to support tumor metastasis, the correlation between proline metabolism reprogramming and organ-specific metastasis remains to be further studied.

## 6 Asparagine metabolism

Asparagine is a NEAA. However, due to the interaction between asparagine and glutamine metabolism, the limited availability of glutamine makes asparagine a conditional essential amino acid. Asparagine can rescue cell proliferation without catabolism in the absence of glutamine. In fact, under the culture condition lacking glutamine, the asparaginase in cells can inhibit the growth and survival of cells, and seriously affect the growth of tumor xenografts *in vivo*. Therefore, in mammals, asparaginase expression is absent in cancer cell lines, which means that mammalian cells generally lack the ability to decompose asparagine into aspartate and free ammonia. This evolutionary adaptability makes cancer cells only use asparagine for protein synthesis, rather than other amino acids or biosynthetic intermediates, making uptake of exogenous asparagine crucial for cancer cell survival and growth (146). In addition to exogenous uptake, endogenous asparagine can be generated from aspartate by asparagine synthetase (ASNS), which is widely expressed in mammalian cells. This ATP-dependent reaction employs glutamine as the nitrogen donor (**Figure 1**) (147).

Aspartate metabolism has been proved to induce tumor metastasis. The aspartate synthetase ASNS is a focus of clinical attention, because the imbalance of the expression of ASNS in children’s acute lymphoblastic leukemia (ALL) cells is considered to offset the impact of a first-line therapy for ALL that depletes asparagine (148–150). It was reported that knocking down ASNS, or limiting asparagine uptake could reduce the bioavailability of asparagine of cancer cells to prevent the induction of mesenchymal gene expression mode, thereby reducing lung metastasis with no obvious effect on the growth of primary breast tumors in mice (151). Moreover, in CRC cells, decreased aspartate biosynthesis could lead to apoptosis of KRAS mutant CRC cells, and reduce cell proliferation and lung metastasis (152). In addition, overexpression of SOX12 promoted asparagine synthesis by transactivating GLS, GOT2, ASNS, thereby promoting proliferation and metastasis of CRC cells (153). These findings support that ASNS has the potential to become a novel tumor biomarker and therapeutic target.

## 7 Discussion

Various studies have provided evidence that adaptive regulation of amino acid metabolism plays an important role in the survival and proliferation of metastatic cancer cells under the metabolic pressure of metastatic microenvironment. The demand for increased energy and biosynthesis usually leads to an increase in the dependence of metastatic tumors on amino acids and related enzymes in their metabolic pathways compared with the primary site. In the microenvironment of metastases, amino acid depletion caused by rapid synthesis, especially glutamine depletion, is one of the main metabolic pressures of the metastatic cells (154). Therefore, the increase of amino acids uptake and NEAAs synthesis can be generally observed in the metastatic cells, which determines the metabolic reprogramming tendency of the metastatic cells compared with the primary site.

It is worth noting that the metabolic reprogramming of metastatic cells is not completely determined by the types of primary tumors, but also depends to some extent on the microenvironment of the metastatic sites, such as the supply of biosynthetic materials or cell signals (155). The microenvironment of different metastatic sites selects cancer cells with specific metabolic advantages, indicating the metabolic heterogeneity of tumor metastasis. Actually, since Stephen Page’s hypothesis of metastasis with the analogy of “seeds” and “soil” in 1889 (156), researchers have found many biomarkers of organ-specific metastasis. For example, studies have shown that exosomes from lung, liver and brain cancer cells in mice and humans fuse with cells in the target organ and participate in the establishment of the pre-metastatic niche, and the exosome integrin can be used to predict organ-specific metastasis (157). Furthermore, the high expression of GABA-related proteins, including GABA receptor, GABA transporter, GABA aminotransferase and glutamic acid decarboxylase, was found in brain metastatic tumor samples of HER2+ and triple-negative breast cancer (158). On this basis, many biomarkers have also been found according to the metabolic characteristics of the metastatic site, such as serine metabolism in the formation of osteoclasts. Compared to breast cancer cells with low bone metastatic activity, the expression of serine metabolic enzymes (including PHGDH, PSAT1 and PSPH) was increased in breast cancer cells with high bone metastatic activity (113). In addition, because lung metastasis is exposed to oxidative stress, the expression of the cystine transporter xCT was observed to be upregulated in lung metastatic breast cancer cells to increase GSH synthesis (36). As more and more studies demonstrate the relationship between organ-specific metastasis and metabolic reprogramming induced by the organ-specific microenvironment, the development of biomarkers and therapeutic targets for organ-specific metastasis has also become a promising direction in tumor research. Although there have been many studies on the driving role of amino acid metabolic reprogramming in organ-specific metastasis, its specific and systematic mechanism still needs further investigation.

Metastasis is the final stage of cancer development. In many kinds of tumors, for patients who have progressed to the stage of metastasis, the current treatment strategy cannot achieve good treatment effect in most cases, which is often related to the drug resistance of the tumor. At the same time, some drug resistance mechanisms may also make the tumor more capable of metastasis (159). For example, in lung adenocarcinoma, overexpression of PSAT1 may lead to tumor metastasis and erlotinib resistance (104, 104). In addition, because the current treatment strategy for tumor metastasis does not make adjustments for the different occurrence of metastasis, but adopts an identical strategy, which may be an important factor leading to drug resistance. For example, some researchers found that disseminated tumor cells remains static in bone marrow, which explains why cytotoxic therapy cannot treat breast cancer patients, and 15-20% of patients may have residual tumor cells after completing auxiliary cytotoxic and endocrine therapy (160, 161). Therefore, the study of metabolic heterogeneity of tumor metastases in different organs can help us to identify clinically available metastasis-related markers and therapeutic targets driven by organ specific microenvironment. In addition, the study of tumor metabolic characteristics can also help us refine the molecular typing of tumors, put forward higher and more refined requirements for tumor treatment strategies, and promote the development of clinical oncology towards personalized cancer medicine.

As previously stated, glutamine is the largest consumed amino acid in rapidly synthesized cells. Glutamine participates in a variety of cellular processes, such as biosynthesis, energy supply, and redox homeostasis as a common nitrogen source. In addition, the transamination of glutamate is also an essential link in the metabolism of amino acids due to its participation in the biosynthesis of nonessential amino acids. The high level of glutamine maintained in plasma can support the metabolic needs of somatic cells, but it is far from enough for metastatic cells. In order to maintain the level of glutamine in the metastatic cells, the glutamine transporters in the metastatic cells are often highly expressed, supporting to grab the glutamine in the microenvironment with the surrounding cells. At the same time, the activation of enzymes in the glutamine metabolism pathway has also been proved to be closely related to the metastasis, not only enhancing the intracellular biosynthesis, but also affecting the cell signal of the metastatic process. For example, GDH1 promotes anoikis resistance and metastasis formation of tumor cells by activating AMPK pathway (61). Because of the close relationship among amino acid metabolism, this specific metabolic advantage is often manifested as a cascade effect of demand. For example, metastatic cancer cells consume a large amount of glutamine to maintain the loss of TCA cycle, and then the dependence of cells on asparagine increases, making inhibition of ASNS sufficient to induce rapid apoptosis of metastatic cancer cells (162). Metabolic demand of amino acids eventuates the metastatic tendency of tumor, reflects metabolic vulnerability and metabolic dependence of metastatic sites, which may provide potential treatments to arrest metastatic seeding and growth.

## Author contributions

ZW and XW wrote the manuscript. KW and H-NC contributed to the conceptualization and supervision of the manuscript. All authors contributed to the article and approved the submitted version.
